# Natural antimicrobial peptide complexes in the fighting of antibiotic resistant biofilms: *Calliphora vicina* medicinal maggots

**DOI:** 10.1371/journal.pone.0173559

**Published:** 2017-03-09

**Authors:** Natalia Gordya, Andrey Yakovlev, Anastasia Kruglikova, Dmitry Tulin, Evdokia Potolitsina, Tatyana Suborova, Domenico Bordo, Camillo Rosano, Sergey Chernysh

**Affiliations:** 1 Laboratory of Insect Biopharmacology and Immunology, Faculty of Biology, St. Petersburg State University, St. Petersburg, Russia; 2 Research Center of Kirov Military Medical Academy, St. Petersburg, Russia; 3 IRCCS AOU San Martino IST - National Institute for Cancer Research, Genova, Italy; Nanyang Technological University, SINGAPORE

## Abstract

Biofilms, sedimented microbial communities embedded in a biopolymer matrix cause vast majority of human bacterial infections and many severe complications such as chronic inflammatory diseases and cancer. Biofilms’ resistance to the host immunity and antibiotics makes this kind of infection particularly intractable. Antimicrobial peptides (AMPs) are a ubiquitous facet of innate immunity in animals. However, AMPs activity was studied mainly on planktonic bacteria and little is known about their effects on biofilms. We studied structure and anti-biofilm activity of AMP complex produced by the maggots of blowfly *Calliphora vicina* living in environments extremely contaminated by biofilm-forming germs. The complex exhibits strong cell killing and matrix destroying activity against human pathogenic antibiotic resistant *Escherichia coli*, *Staphylococcus aureus* and *Acinetobacter baumannii* biofilms as well as non-toxicity to human immune cells. The complex was found to contain AMPs from defensin, cecropin, diptericin and proline-rich peptide families simultaneously expressed in response to bacterial infection and encoded by hundreds mRNA isoforms. All the families combine cell killing and matrix destruction mechanisms, but the ratio of these effects and antibacterial activity spectrum are specific to each family. These molecules dramatically extend the list of known anti-biofilm AMPs. However, pharmacological development of the complex as a whole can provide significant advantages compared with a conventional one-component approach. In particular, a similar level of activity against biofilm and planktonic bacteria (MBEC/MIC ratio) provides the complex advantage over conventional antibiotics. Available methods of the complex *in situ* and *in vitro* biosynthesis make this idea practicable.

## Introduction

Bacteria exist in two basic life forms: planktonic form destined for bacteria proliferation and biofilms enabling them to survive in adverse conditions. Biofilms, sedimented microbial communities embedded in a biopolymer matrix have come to particular prominence since they cause main part of human bacterial infections and highly resistant to antibiotics and host immunity (for review see [1–5]). Recent studies have found that bacterial biofilms play a role in the onset and development of stomach [6], colorectal [7], colon [8], prostate [9], breast [10] and lung [11] cancer. Thus, biofilms’ impact on the human life and death extends far beyond the classic field of infectious pathology. This makes the search for new anti-biofilm drug candidates especially relevant. Naturally occurring antimicrobial peptides (AMPs) are generally recognized as a prospective platform for antibacterial drugs development [12–16]. Although virtually all studied to date multicellular animals use some form of AMPs to defend against pathogens, their greatest diversity is found in insects. Classification, structural and functional diversity and mechanism of action of insect AMPs are considered in review articles [17, 18]. Considerable attention was paid to insect AMPs as potential drugs [18–21]. However, therapeutic AMPs development meets a bunch of well-known obstacles [22–24]. Among other things should be noted that the development focuses on the individual antimicrobial compounds capable of stopping the infection when applied alone. This approach corresponds well to the experience of conventional antibiotics development. In contrast, natural AMP-based defense systems use complexes of active molecules rather than single molecule approach [25]. The benefits of multicomponent AMP systems for the host remain poorly understood although the synergistic/potentiating actions among naturally co-occurring AMPs [26–29] and the capacity to prevent resistance development in bacteria [30] were described in experiments with various forms of planktonic bacteria.

This article analyses anti-biofilm activity of host AMP complex, which remains unexplored though some individual AMPs under review [15, 31–33] demonstrated activity against biofilms. Based on our previous work, we have chosen for experimental study naturally occurring AMP complex isolated from the maggots of blowfly *Calliphora vicina* (Diptera, Calliphoridae) and named here FLIP7 (abbreviation from Fly Larvae Immune Peptides 7). Like other members of Calliphoridae family known in medicine as a "medicinal maggots” [34], *C*. *vicina* maggots in response to bacterial infection immediately synthesize and accumulate in the hemolymph four families of cationic AMPs: defensins, cecropins, diptericins and proline-rich peptides [30, 35, 36]. The hemolymph also contains other immune response related compounds including antiviral and antitumor peptides alloferons [37]. Calliphora defensin, as well as defensins of other insects and vertebrates, is a peptide with a 3D structure containing α-helix/β-sheet elements coordinated by 3 disulfide bridges and is predominantly active against Gram-positive bacteria. Mode of insect defensins’ antibacterial activity comprises bacterial cell wall disruption/permeabilization [17]. Calliphora cecropin is a linear amphipathic α-helical peptide particularly active towards Gram-negative bacteria. All insect cecropins are known to have pore forming and cell membrane permeabilizing activity [17]. Calliphora diptericins are members of a glycine-rich AMP family selectively toxic to some Gram-negative Enterobacteria like *E*. *coli* by means of cell wall disruption [38]. Calliphora proline-peptides [30, 36] belong to the family of proline/arginine-rich AMPs, which is known to kill bacteria by interfering DNA and/or protein synthesis [39].

## Materials and methods

### Insects

Experiments were performed with a laboratory colony of blue blowfly *C*. *vicina* originating from St. Petersburg area (North-West of Russia) and characterized by stable larval diapause. Breeding conditions were essentially the same as previously described [40]. To induce diapause in the progeny, adult flies were kept under short day conditions (12L:12D). The larvae were fed by fresh beef at 12°C, third instar larvae were transferred to 3°C at the end of feeding period, left there for 2 weeks to form diapause and then taken to the experiments.

### *C*. *vicina* AMP complex preparation

The process of the complex preparation was the same as described [30]. Lyophilized live culture of *E*. *coli* M17 strain (Microgen, Russia) suspended in sterile distilled water was used to induce an immune response in diapausing *C*. *vicina* larvae. The larvae were pricked with a needle previously dipped into the suspension containing 10^10^ bacterial cells/mL and were left overnight at 25°C. Their surface was then sterilized in 70% ethanol, washed with sterile distilled water and dried. Hemolymph (approximately 10 μl per animal) was collected in ice-cold tubes through a cuticle puncture. Hemolymph samples were kept at -70°C until use. Thawed hemolymph was acidified with 0.1% trifluoroacetic acid (TFA) to a final concentration of 0.05% and insoluble particles were removed by centrifugation (30 min at 8000g at 4°C). The supernatant was applied to reversed-phase Sep-Pak C18 cartridges (Waters) stabilized by 0.05% TFA in the amount of 5 mL/g of sorbent. Highly hydrophilic compounds were removed by cartridge washing with 0.05% TFA. Compounds absorbed in the cartridge were eluted with 50% acetonitrile solution acidified with 0.05% TFA, lyophilized (FreeZone, Labconco) and stored at -70°C. Prior to use, the lyophilized sample was dissolved in deionized water (50 mg/mL), sterilized by filtration through a membrane with a pore size 0.22 μm (Milliex-GS, Millipore) and frozen at -70°C.

### Antimicrobial peptides characterization

The complex AMPs were previously isolated and characterized by a combination of reversed phase HPLC, MS and Edman degradation methods [25, 35]. In that work 1 mg of the lyophilized compound was dissolved in deionized water and applied to Shimadzu LC20 Prominence HPLC system equipped with analytical column C18 Vydac (4.6 × 250 mm, 5 μm, Grace), equilibrated with 0.05% TFA. The column was eluted with a linear gradient of acetonitrile (ACN) from 0 to 50% in acidified water (0.05% TFA) for 50 min. Chromatographic fractions were automatically collected with 1 min intervals. The fractions’ optical densities were registered by means of a UV detector at two fixed wavelengths 214 and 280 nm. The fractions were lyophilized, dissolved in deionized water and tested against planktonic *M*. *luteus* A270 and *E*. *coli* D31strains using the plate growth inhibition assay. Anti-biofilm activity of the fractions against one-day biofilms formed by E. coli ATCC 25922 и S. aureus 203 was analyzed by TTC and crystal violet assays as described below.

### Mass spectrometry

The chromatographic fractions containing antibacterial materials were diluted in deionized water to 100 μL volume. The molecular masses of the materials were determined by the MicroTOF ESI method on a MaXis chromato-mass spectrometer (Bruker Daltonik, Bremen, Germany). Mass specters were registered using positive-ion mode in 50–1000 mass diapasons. Capillary voltage was established at 4500 V, and 500 V at the end of the capillary. Dry gas was applied at flow rate 4 L/min under temperature 180°C. In-source collision-induced dissociation (ISCID) was turned on, and collision voltages up to 200 V were used. Mass spectrograms were performed manually to take into account that all the peaks differ from the background signal. Experimentally determined masses were compared with the previously published characteristics of *C*. *vicina* individual AMPs.

### Transcriptome analysis

An immune response leading to the sharp activation of AMPs biosynthesis was induced in diapausing larvae 1 week after the end of the feeding period in accordance with the procedure described in the section “C. vicina AMP complex preparation”. After 18 hours, when the AMPs titer reaches a maximum in the hemolymph [35], the larvae were frozen in liquid nitrogen and ground. Isolation of total RNA was performed using TRIzol^®^ Reagent according to the manufacturer's protocol (https://tools.thermofisher.com/content/sfs/manuals/trizol_reagent.pdf). The resulting total RNA was used for cDNA synthesis using the Roche cDNA synthesis system (https://lifescience.roche.com/shop/products/cdna-synthesis-system). cDNA was sequenced on the device Ion Torrent and cDNA library was created using software Mira. Search of antimicrobial peptide sequences in the cDNA library was done using Blast software and previously published *C*. *vicina* AMPs as queries. Alignment method: compositional matrix adjust [41]. In the analysis were used sequences with 70–100% similarity (P <0.001).

### Antimicrobials

The following commercial preparations were used as reference antibiotics in the experiments: meropenem trihydrate from carbopenems (AstraZeneca), third generation cephalosporin sodium cefotaxime (Abolmed), naturally occurring polypeptide polymyxin B sulfate (Applichem) and glycopeptide vancomycin (Kraspharma). The antibiotics were dissolved in sterile deionized water in concentration 1 mg/ml, aliquoted in 0.05 ml volumes and kept at -70°C until use.

### Bacteria

*Escherichia coli* ATCC 25922 and NCTC 13353 strains as well as a series of clinical strains of *Staphylococcus aureus* and *Acinetobacter baumannii* have been used as model biofilm forming bacteria. The annotated genome assembly of *E*. *coli* ATCC 25922 and NCTC 13353 strains are available under the links http://www.ncbi.nlm.nih.gov/nuccore/CP009072 and http://www.ebi.ac.uk/ena/data/view/ERS530434 respectively. *A*. *baumannii* 28 genome is sequenced and deposited in Genbank (Acc. № NZ_MAFT00000000.1) and available under the link https://www.ncbi.nlm.nih.gov/nuccore/NZ_MAFT00000000. The strain genome sequencing predicts resistance to aminoglycosides, beta-lactams and chloramphenicol. The clinical strains originate from the surgery clinic of the Kirov Military Medical Academy (St. Petersburg, Russia), except for the strains of *A*. *baumannii* 28 and *S*. *aureus* 203 originating from the collection of Northwestern State Medical Mechnikov University and Institute of Experimental Medicine (St. Petersburg, Russia), respectively. *E*. *coli* D31 and *Micrococcus luteus* A270 strains routinely employed in insect AMP studies were obtained from the Institute of Genetics and Molecular and Cellular Biology (France) and used for the complex AMPs characterization. Profiles of the clinical strains’ antibiotic resistance were determined as recommended (National Committee for Clinical Laboratory Standards, 2003. Performance Standards for Antimicrobial Disk Susceptibility Tests. Approved standard M2-A8. NCCLS, Wayne, PA). The strains were classified as susceptible, intermediate or resistant to the antibiotic tested by disc diffusion method. To test resistance profiles of *A*. *baumannii* strains the following antibiotics were used: amikacin, gentamicin, imipenem, meropenem, cefepime, cefoperazone, cefotaxime, ceftazidime, ciprofloxacin (Oxoid, Great Britain). The strain 143 was found resistant to all antibiotics tested except amikacin, which was intermediately effective. The strain 149.2 demonstrated resistance to cefoperazone, cefotaxime, ceftazidime, intermediate resistance to cefepime and sensitivity to the rest of antibiotics. *S*. *aureus* profiles were tested against amikacin, penicillin, vancomycin, gentamicin, doxycycline, azithromycin, clindamycin, chloramphenicol, netilmicin, oxacillin, cefazolin, ciprofloxacin, fusidic acid, linezolid (Oxoid, Great Britain). The strain 203 was found sensitive to all tested antibiotics while strain 73.1 showed resistance to azithromycin only. Moreover, serial microdilution tests demonstrated sensitivity to polymyxin B in all *A*. *baumannii* and *E*. *coli* strains used in experiments.

### Antibacterial activity assays

#### Solid growth inhibition assay

Standard plate-growth inhibition assay was employed for identification and relative quantification of the complex active compounds. The method was essentially the same as the one previously described [30]. *E*. *coli* D31 and *M*. *luteus* А270 cultures were grown in LB liquid nutrient medium (Invitrogen) for 18–20 hours at 37°C. Sterile Petri dishes (9 cm in diameter) were filled with 7.5 mL of LB medium supplemented with 12g/L agarose (Invitrogen). 4 x 10^6^ CFU/dish test microorganisms measured by OD were inoculated into the warm medium. The analytes were dissolved in 20 μl of deionized sterile water and 2 μl aliquot of the solution was applied onto a solid medium surface. The diameter of the growth inhibition zone was measured after 24-hour incubation at 37°C and the inhibition zone area was calculated and used for relative quantification of the AMP anti-*M*. *luteus* and anti-*E*. *coli* activity.

#### Liquid growth inhibition assay

The standard microdilution method was carried out for MIC determination with LB broth (Invitrogen), as recommended (Clinical Laboratory Standards Institute, 2015. Methods for dilution antimicrobial susceptibility tests for bacteria that grow aerobically. Approved Standard, 10^th^ edition, M07-A10. CLSI, Wayne, PA). Briefly, individual wells of a 96-well tissue culture plate (Sarstedt, Newton, NC) containing 100 μl of liquid nutrient medium LB (Invitrogen) with doubling antibiotic dilutions were inoculated with approximately 5 x 10^5^ CFU/mL of test bacteria. The initial inoculum was grown on the solid LB agar nutritive medium (Invitrogen), individual colonies were picked up, transferred into liquid medium (Luria broth base, 25 g/L) and incubated overnight at 37°C. Microtiter plates were incubated for 20 hours at 37°C.

### Biofilm formation

Preparation of 24-h old biofilm corresponds to the procedures outlined by Christensen *et al*. [42]. In brief, bacteria were cultured in 5 ml of LB liquid nutrient medium (Invitrogen) for 18–20 hours at 37°C. Overnight cultures were adjusted 5x10^5^ CFU/ml test microorganisms measured by OD. One hundred microliter aliquots of the diluted bacterial suspension were inoculated into each well of a 96-well flat-bottomed polystyrene plate (Sarstedt AG & Co., Newton, NC) and incubated in a humidified incubator for 24 h at 37°C. The negative control was LB liquid nutrient medium.

Crystal violet assay has been employed to visualize and quantify biofilm formation capacity of bacteria as recommended [43]. Bacteria were incubated 24 h in the 96-well plates, washed out of culture medium and stained by crystal violet as described in the section Biofilm eradication assay. Optical density in the well has been used as a measure of biofilm thickness. Each experiment was performed in 8 replicates.

### Biofilm microscopic visualization

Coverslip 24x24 mm (Thermo Fisher Scientific Gerhard Menzel B.V. & Co. KG, Braunschweig, Germany) was placed in each well of 6-well tissue culture microtitre plate (Falcon, Becton Dickinson Labware, Franklin Lakes, NJ, USA). The wells were filled with 2.8 mL of bacterial suspension prepared as mentioned above and the plates were incubated 24 hours at 37°C. Then the 24-hour biofilms were washed three times with 3 mL of sterile physiological buffered saline (PBS) to remove unattached bacteria and filled with fresh culture medium (control) or the medium supplemented with 4 mg/mL of FLIP7. The plates were incubated for another 24 hours at 37°C. Then coverslips were removed from the wells, washed in PBS, placed on a glass slide upside down and photographed through a Leica DMI 2500 microscope (Leica Microsystems GmbH, Wetzlar, Germany), Nomarski optics x 400 and x 1000.

### TTC cell killing assay

We used colorimetric antimicrobial susceptibility test which can measure the antimicrobial susceptibility of biofilmed bacteria [44]. This method uses trimethyl tetrazolium chloride (TTC) as an indicator of viable bacteria and is comprised of the following steps: preparation of a 24-h old biofilm, treatment with the antimicrobial agent, addition of 0.02% TTC and measurement of TTC absorbance at 540 nm. Since reduction of TTC by viable bacteria produces red formazan, bacterial growth inhibition can be measured quantitatively by colorimetric absorbance at 540 nm. The 24-h biofilms in a 96-well tissue culture microtitre plates were washed three times with 200 μL of sterile physiological buffered saline (PBS) solutions to remove unattached bacteria and air-dried. Serial two-fold dilutions of antimicrobials were prepared. 100 μL of each concentration was added to each corresponding well and plates were incubated 24 h at 37°C in a humidified incubator. After incubation, 11 μl of 0.2% TTC (Lenreactive, Russia) was added to a final concentration of 0.02%. After 1 h incubation at 37°C, the OD_540_ was measured on the Epoch microplate reader (BioTek). The mean OD_540_ value of 48-h biofilmed cells without treatment with the antimicrobial agent was set as the control. Each experiment was performed in triplicate. Then, the minimum biofilm inhibitory concentration (MBIC) was determined as the lowest concentration of drug that resulted in a mean % value less than 100%.

### Crystal violet biofilm eradication assay

Biofilm eradication assays were performed using a previously described method with some modifications [43]. The 24-h biofilms in a 96-well tissue culture microtitre plates were washed three times with 200 μL of sterile PBS solutions and air-dried. Serial two-fold dilutions of antimicrobials were prepared. 100 μL of each concentration was added to each corresponding well and plates were incubated 24 h at 37°C. After the incubation, the waste media was removed and plates were washed three times with 200 μL PBS solutions, air-dried and stained with crystal violet 0.1% (in water) (Lenreactiv, Russia) for exactly 2 min. The stained biofilms were washed three times with 200 μL PBS solutions, air-dried and solubilised with 200 μL 95% ethanol for 1 h. Then the biofilm cell-associated dye was measured at OD_570_ using the Epoch reader (BioTek). Each experiment was performed in triplicate. The minimum biofilm eradication concentration (MBEC) was assessed with MBEC_50_ and MBEC_90_, which is the concentration of drugs decreasing crystal violet binding in preformed biofilms, by 50% and 90%, respectively.

### Cytotoxicity assay

Human mononuclear leukocytes (lymphocytes and monocytes) have been used as a model for FLIP7 cytotoxicity assessment. The cells were isolated from donor venous blood by means of BD Vacutainer^®^ CPT (BD Biosciences) as recommended by the manufacturer, and transferred to RPMI culture medium enriched with glutamine, 10% fetal calf serum and antibiotic antimycotic solution A5955 (Sigma-Aldrich). Lyophilized FLIP7 was diluted in the culture medium, passed through the filter 0.45 μm and introduced into the medium a final concentration 0.5, 5 and 10 mg/mL. Then, the number of lymphocytes and monocytes differentially stained with annexin V (apoptosis marker) and propidium iodide (PI, necrosis marker) was counted using a standard flow cytometry Annexin-V-FITC binding assay according to the manufacturer’s instructions (BioRad ANNEXIN V KIT). Analyses were performed by BD FACSAria III flow cytometer (530/30nm and 616/23nm filters) with BD FACSDiva software. The cells were divided into the following categories:

- Normal (undamaged) cells (annexin V and PI double negative)
- Apoptotic (annexin V positive)
- Necrotic (annexin V negative, PI positive)

Each experiment included 6 independent replications. Average numbers of apoptotic and necrotic cells per 100 gated cells were used as a quantitative indicator of FLIP7 toxicity.

### Statistical methods

MIC, MBIC and MBEC values (mean ± standard error) were calculated by means of the Primer of Biostatistics software, version 4.03. Statistical significance of the differences was analyzed by ANOVA considering P values <0.05 as significant.

## Results

### Biofilm forming capacity of tested bacteria

To visualize and quantify the ability of bacteria to form biofilms, the standard crystal violet staining method has been employed. All the studied bacteria were able to form detectable biofilms on the plastic surface, but a one-day biofilm thickness measured in units of crystal violet optical density varied from strain to strain (Table 1). Maximal ability to form biofilms was detected in *E*. *coli* ATCC 25922 and *S*. *aureus* 203 strains.

**Table 1:** Biofilm formation capacity of tested bacteria (crystal violet assay).

Bacteria	Strain characteristics	Optical density A_570_ nm, units		P*	Biofilm formation capacity
		Control	1 day biofilm		
E. coli ATCC 25922	Antibiotic sensitive	0.1 ± 0.002	0.7 ± 0.05	<0.001	High
E. coli NCTC 13353	Beta-lactamase producing	0.1 ± 0.004	0.1 ± 0.03	0.17	Low
S. aureus 203	Antibiotic sensitive	0.1 ± 0.002	1.1 ± 0.08	<0.001	High
S. aureus 73.1	Azitromycin resistant	0.1 ± 0.01	0.3 ± 0.03	0.03	Intermediate
A. baumannii 28	Antibiotic multiresistant	0.1 ± 0.002	0.6 ± 0.04	<0.001	High
A. baumannii 143	Antibiotic multiresistant	0.1 ± 0.01	0.26 ± 0.03	0.02	Intermediate
A. baumannii 149.2	Antibiotic multiresistant	0.1 ± 0.01	0.8 ± 0.09	0.002	High

*P–statistical significance of differences in optical density compared to control wells without added bacteria. Each test consisted of 3 independent measurements.

### Microscopic visualization of biofilm formation and FLIP7 anti-biofilm effect

**Fig 1:**
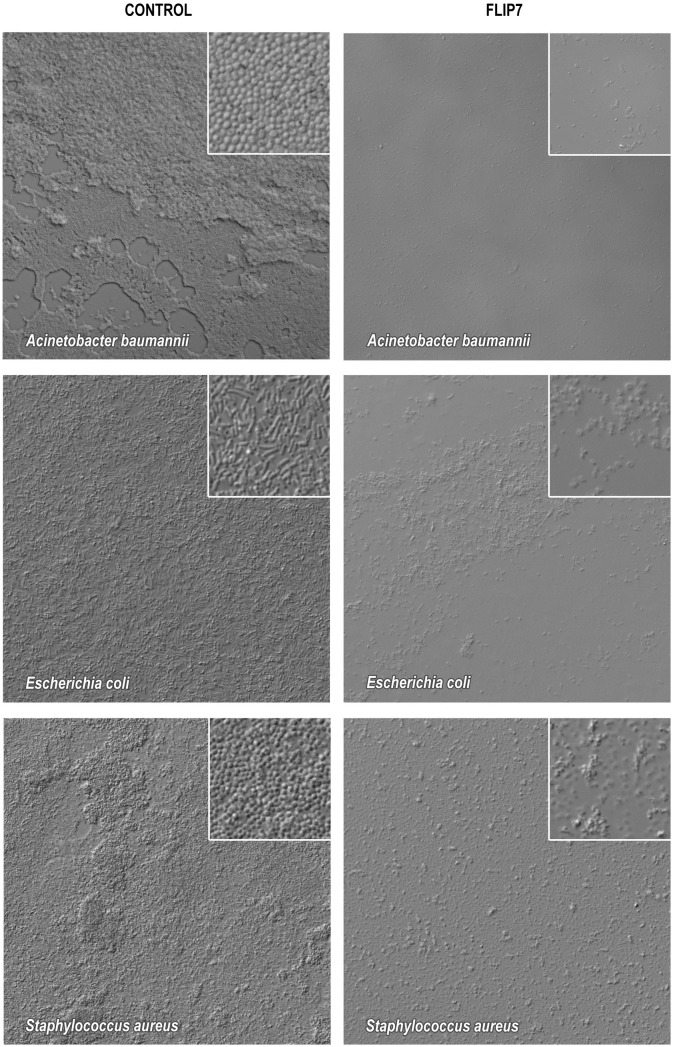
Photomicrographs of biofilms formed by three bacterial strains in normal conditions and in the presence of FLIP7. *A*. *baumannii* 28, *E*. *coli* ATCC 25922 and *S*. *aureus* 203 biofilms grown as described in Materials and Methods section were incubated 24 hours in the culture medium (control) or the medium supplemented with 4 mg/mL of FLIP7 and photographed using Nomarski optics at 400-fold and 1000-fold (inset) magnification. All three strains formed dense biofilms containing live bacteria attached to the glass surface (control). At the same time, FLIP7 presence in the medium led to the destruction of the biofilm, in the remains of which are visible mainly cells that have lost their characteristic shape.

The biofilm formation was also analyzed by light microscopy using *A*. *baumannii* 28, *E*. *coli* ATCC 25922 and *S*. *aureus* 203 strains, which demonstrated high biofilm formation capacity in crystal violet assay. All three strains formed dense biofilms containing live bacteria attached to the glass surface (Fig 1). The appearance of FLIP7 treated biofilms differed sharply from that of the normal state. Remains of biofilms turned into formless detritus and the few remaining bacterial cells resembled the cells treated with membrane-acting peptides [45, 46].

### The impact of FLIP7 and conventional antibiotics on the viability of planktonic cultures and biofilms

Comparison of the sensitivity of bacteria living in planktonic and biofilm forms to FLIP7 and reference antibiotics has been made by means of standard TTC cell viability assay (Table 2). Reference antibiotics were selected for each strain based on its antibiotic resistance profile described in Materials and Methods section. All tested materials demonstrated anti-biofilm activity quantitatively characterized by MBIC_90_ value. However, MBIC_90_/MIC ratio demonstrates the resistance growth variation depending on the biofilm-forming bacteria features. In *E*.*coli* strains resistance to FLIP7 grew from 3.3 to 10.3 times (6.8 ± 3.5 in average) whereas resistance to reference antibiotics increased 24–40 (31.4 ± 3.4) times. In *S*. *aureus* biofilms FLIP7 resistance growth was even less significant: 0.9–2.3 times (1.6 ± 0.7) as compared to planktonic forms while meropenem resistance growth varied in broad range from 3.6 to >167 times. Resistance in *A*. *baumanii* biofilms grew from 18 to 51 times (33 ± 9.64 in average) to FLIP7 and 43 to 667 times to polymyxin B. Since both polymyxin B and FLIP7 active compounds belong to AMPs, this bacteria seems to form biofilms well adapted to AMPs attack in contrast to *E*. *coli* and *S*. *aureus*. It is notable that beta-lactam antibiotic meropenem demonstrated stronger anti-biofilm activity against *A*. *baumannii* and *S*. *aureus* 73.1 but not against other bacteria.

**Table 2:** Planktonic and biofilm bacteria sensitivity to FLIP7 and reference antibiotics (TTC assay).

Strain	Planktonic bacteria MIC, μg/mL	Biofilm bacteria MBIC_90_, μg/mL	P*	MBIC_90_/ MIC ratio
*E*. *coli* ATCC 25922				
FLIP7	500 ± 0.00	1667 ± 88	<0.001	3.3
Cefotaxime	0.125 ± 0.00	3 ± 0.7	0.005	24
Meropenem	0.03 ± 0.00	0.9 ± 0.3	<0.001	30
Polymyxin	0.5 ± 0.00	12.3 ± 0.3	<0.001	24.6
*E*. *coli* NCTC 13353				
FLIP7	250 ± 0.00	2567 ± 617	0.02	10.3
Meropenem	0.06 ± 0.00	2.3 ± 0.9	0.073	38.3
Polymyxin	1.3 ± 0.3	52 ± 14	0.022	40.0
*S*. *aureus* 203				
FLIP7	667 ± 167	1543 ± 137	0.015	2.3
Meropenem	0.03 ± 0.005	>5		>167
Vancomycin	0.5 ± 0.00	25± 13	0.134	50
*S*. *aureus* 73.1				
FLIP7	8000 ± 0.00	7333 ± 167	0.016	0.9
Meropenem	0.08 ± 0.00	0.29 ± 0.08	0.06	3.6
*A*. *baumannii* 28				
FLIP7	125 ± 0.00	6433 ± 1417	0.011	51
Polymyxin B	0.4 ± 0.1	267 ± 67	0.016	667
*A*. *baumannii* 143				
FLIP7	125 ± 0.00	3767 ± 33	<0.001	30
Polymyxin B	0.25 ± 0.00	>20		>80
*A*. *baumannii* 149.2				
FLIP7	416 ± 83	7600 ± 306	<0.001	18
Meropenem	0.5 ± 0.00	1.5 ± 0.06	<0.001	3
Polymyxin B	0.7 ± 0.2	30.3 ±6.2	0.009	43

*Each test consisted of 3 independent measurements

### FLIP7 and reference antibiotics comparative cell killing and biofilm eradicating activities

Anti-biofilm activity of the materials was further studied by means of TTC and crystal violet assays using *E*. *coli* ATCC 25922 and *S*. *aureus* 203 strains as models (Table 3). *E*. *coli* biofilms demonstrated 6-time resistance growth to FLIP7 in comparison with planktonic culture according to both cell viability TTC and biofilm eradicating crystal violet assays. Thus, these two FLIP7 effects realized at the same concentration and with this point of view, they are closely related. Cefotaxime and meropenem reduced biofilm thickness by 90% when applied in a concentration 4 times greater than planktonic culture MIC, but it did not lead to bacteria death. To kill 90% of bacteria it was necessary to increase their concentration in the 17–33 times. Polymyxin concentration destroying 90% of the bacteria in *E*. *coli* biofilm exceeded the MIC of planktonic culture 50 times.

**Table 3:** FLIP7 and reference antibiotics effects on the *E*. *coli* ATCC 25922 and *S*. *aureus* 203 biofilms cell viability and thickness.

	Planctonic	Biofilm cells viability*			Biofilm thickness*		
Strains	MIC*	(TTC assay)			(Cristal violet assay)		
	μg/mL	MBIC_90_	MBIC_90/_	MBEC_50_	MBEC_50_/	MBEC_90_	MBEC_90_/
		μg/mL	MIC	μg/mL	MIC	μg/mL	MIC
*E*. *coli*							
FLIP7	667 ± 167	4100 ± 208	6.15	817 ± 60	1.2	4083± 1543	6.1
Cefotaxime	0.125 ± 0.00	4.17 ± 0.83	33.4	0.14 ± 0.03	1.1	0.53 ± 0.04	4.24
Meropenem	0.06 ± 0.00	1 ± 0.00	16.7	0.14 ± 0.01	2.3	0.24± 0.0006	4.0
Polymyxin	0.5 ± 0.00	24.7 ± 0.3	49.4	9.4 ± 0.3	18.8	14.3 ± 1.1	28.6
*S*. *aureus*							
FLIP7	500 ± 0.00	933 ± 17	1.9	590 ± 105	1.2	973 ± 15	1.9
Meropenem	0.03 ± 0.00	>5	>167	3.5 ± 0.8	116.7	>5	>167
Vancomycin	0.6 ± 0.00	>50	>83	17.5 ± 5.1	29.2	>50	>83

*Each test consisted of 3 independent measurements

Experiment with *S*. *aureus* biofilm revealed even more remarkable difference between FLIP7 and reference antibiotics. *S*. *aureus* biofilm resistance against FLIP7 in comparison with planktonic culture grew less than 2 times according to both TTC and crystal violet assays. At the same time the MBEC_90_ values of meropenem and vancomycin exceeded the maximum definable level 167 and 83 times, respectively. Additionally, Table 3 contains MBEC_50_ values demonstrating dramatic growth of *S*. *aureus* biofilm resistance to the reference antibiotics but not to FLIP7.

### Composition of FLIP7 antimicrobial peptides

**Fig 2:**
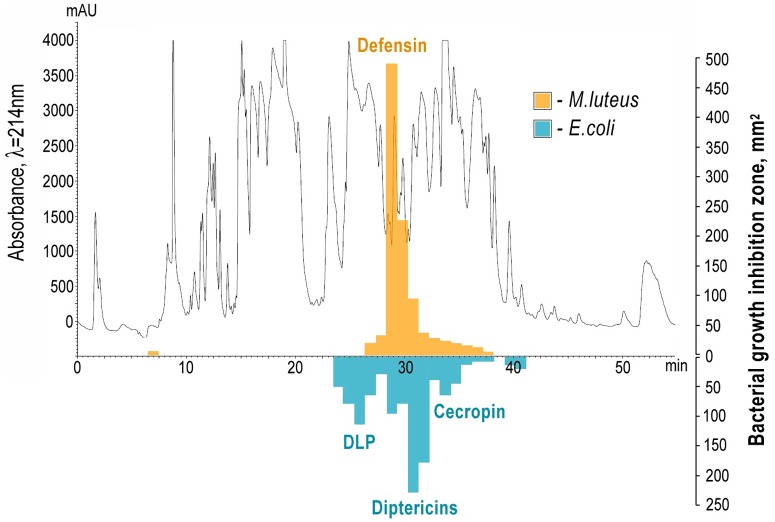
*C*. *vicina* AMP complex chromatographic fractionation and anti-biofilm compounds positioning. The complex was exposed to reversed phase HPLC and the resulting fractions antimicrobial activity against *E*. *coli* D31 and *M*. *luteus* A270 strains was tested using solid growth inhibition assay as explained in Materials and Methods section. The resulting fractions were used for mass spectrometry analysis of the composition and antimicrobial activity of the components. Active fractions are marked by bars with a height corresponding to the area of the growth inhibition zone.

Composition of FLIP7 AMPs was primarily characterized by combination of liquid chromatography and solid growth inhibition assays (Fig 2). 1 mg of FLIP7 was fractionated by HPLC, 52 fractions collected with 1 min intervals were lyophilized and their antibacterial activities were quantified through solid growth inhibition assay using planktonic cultures of Gram-negative *E*. *coli* D31 and Gram-positive *M*. *luteus* A270 bacteria embedded to solid agar medium. The majority of anti-*M*. *luteus* activity was present in fractions 28 to 30, whereas compounds active against *E*. *coli* were found in a broad range of fractions starting from 23 to 34. The active fractions distribution was essentially the same as described previously [30].

Anti-biofilm activity of the same chromatographic fractions was analyzed using combination of TTC and crystal violet assays (Table 4). Fractions 1–21 and 40–52 containing no antibacterial activities against planktonic (solid growth inhibition assay) and biofilm (TTC, crystal violet assays) bacteria are excluded from Table 4 for simplicity. Experiments with *E*. *coli* ATCC 25922 biofilm revealed evidential cell killing activity in fractions 26 and 28 to 33 and biofilm eradicating activity in fractions 28 to 34, respectively. According to the solid growth inhibition assays (Fig 2), in which planktonic bacteria were tested, all fractions can be divided into inactive (no zone of inhibition) and active (the zone size varies depending on the activity of the fraction). The optical density in inactive fractions 5–21, 37, 40–47 (n = 26) measured by TTC and crystal violet assays was, respectively, 0.7 ± 0.016 AU_540_ nm (σ = 0.076) and 1.23 ± 0.045 AU_570_ nm (σ = 0.229). The active fractions include two subsets with respect to anti-biofilm activity. Most of the anti-biofilm activity belongs to the range of fractions 28–33, in which the cell viability and biofilm thickness reach the minimum level. Here the optical densities in TTC and crystal violet assays in each fraction are far beyond 3 σ threshold for the mean value of inactive fractions. Mean values of TTC (0.23 ± 0.067) and crystal violet (0.26 ± 0.047) optical densities in these fractions are also significantly lower (P<0.001) in comparison with inactive fractions. Highest cell killing activity (96% suppression compared to the mean value of inactive fractions) belongs to the fraction 32 whereas highest biofilm eradicating activity (90% eradication) to the fraction 31. At the same time, fractions 22–27 and 38–39 active in solid growth inhibition assay did not show evidential activity against *E*. *coli* biofilm.

**Table 4:** Cell killing (TTC assay) and biofilm eradicating (crystal violet assay) activity of FLIP7 fractions against *E*. *coli* and *S*. *aureus* biofilms.

Fraction №	*S*. *aureus*				*E*. *coli*			
	TTC		Crystal violet		TTC		Crystal violet	
	OD	%*	OD	%*	OD	%*	OD	%*
22	1.28		1.09		0.77		1.29	
23	1.44		0.99		0.84		1.11	
24	1.38		0.85		0.71		0.93	
25	1.54		1.08		0.45	**64**	0.96	
26	1.44		1.14		0.46	**66**	0.74	
27	1.44		0.81		0.70		0.74	
28	**0.08**	**6**	**0.22**	**17**	**0.05**	**7**	**0.17**	**14**
29	**0.12**	**9**	**0.33**	**26**	**0.30**	**43**	**0.24**	**20**
30	0.75		**0.33**	**26**	**0.09**	**13**	**0.14**	**11**
31	1.96		1.07		**0.24**	**34**	**0.12**	**10**
32	1.32		0.57		**0.03**	**4**	**0.29**	**24**
33	1.09		0.62		0.41	**59**	**0.39**	**32**
34	1.21		1.10		0.47		**0.44**	**36**
35	1.23		1.15		0.47		1.26	
36	1.30		1.17		0.47		0.72	
37	1.39		0.56		0.56		0.56	
38	1.53		0.73		0.62		0.73	
39	1.45		1.10		0.62		1.10	

*% of the mean level of inactive fractions

Experiments with *S*. *aureus* 203 biofilm revealed strong cell killing activity in fractions 28 and 29 (94 and 91% suppression compared to inactive fractions, respectively) and biofilm eradicating activity in fractions 28–30 (83 to 74%). The optical density of the inactive fractions in the TTC and crystal violet assays was, respectively, 1.3 ± 0.04 AU_540 nm_ (σ = 0.244) and 1.26 ± 0.04 AU_570 nm_ (σ = 0.259). These values in active fractions are far beyond 3 σ threshold of inactive fractions and cannot be explained by random variation. Comparison of mean values of anti-biofilm active (0.1 ± 0.02 and 0.29 ± 0.037 for TTC and crystal violet assays, respectively) and inactive fractions confirms the statistical significance of these differences (P<0.001). Fractions 26, 27 and 31–37, although they showed residual activity on planktonic culture, have no effect on the state of *S*. *aureus* biofilm.

### Mass spectrometric identification of FLIP7 anti-biofilm compounds

The fractions 26, 28–34 exhibiting anti-biofilm activity in TTC and/or crystal violet assays were further analyzed by electrospray mass spectrometry and the masses obtained were compared with the masses of known *C*. *vicina* AMPs (Table 5). Fractions 25–26 were found to contain the peptide with mass identical to domesticin-like peptide. Fractions 28 and 29 were found to contain, besides other compounds, proline-rich peptide (2987 Da) and defensin (4032 Da) and demonstrated high cell killing and biofilm eradicating activities both against *E*. *coli* and *S*. *aureus* biofilms, particularly fraction 28. Fraction 30 contained two masses corresponding to the masses of previously sequenced *C*. *vicina* diptericin AMPs. This fraction exhibited maximal cell killing and biofilm eradicating activity against *E*. *coli* and moderate biofilm eradicating (but not cell killing) activity against *S*. *aureus*. Fractions 33–34, among other compounds, contained the mass 4156 Da precisely corresponding to the mass of cecropin, previously sequenced *C*. *vicina* AMP. Fractions 31–32 did not contain any known *C*. *vicina* AMPs although demonstrated anti-*E*. *coli* (but not anti-*S*. *aureus*) activities. The fraction 31 contained three groups of materials with masses noted in the Table 5. The fraction 32 consisted of solely one group, which is characterized by masses similar or identical to only one group of the materials from fraction 31. So, fractions 31 and 32 seem to contain a novel group of AMPs. It is notable that fraction 31 demonstrated predominantly cell eradicating activity in contrast to the fraction 32 having mainly cell killing activity. This fact points to the presence of at least two compounds with complementary anti-biofilm activity.

**Table 5:** Mass spectrometric characteristics and activity profiles of FLIP7 anti-biofilm AMPs.

Fraction	AMPs Molecular masses, Da		Relative anti-biofilm activity^1^			
	Found in the sample	Masses of known peptides	*E*. *coli*		*S*. *aureus*	
			TTC	Crystal violet	TTC	Crystal violet
25–26	4442.2	4442.2 (domesticin-like peptide)^2^	**+**	**-**	**-**	**-**
28	2986.7		**+++**	**+++**	**+++**	**+++**
	4032.5	2987.0 (P-rich)^3^				
29	2986.7	4032.0 (defensin)^3^	**+**	**++**	**+++**	**++**
	4032.5					
30	8886.2	8886.2 (diptericin)^3^	**+++**	**+++**	**-**	**++**
	8999.4	8999.7 (diptericin)^3^				
31	3483–3631		**+**	**+++**	**-**	**-**
	6773–6973	Not found				
	8468					
32	6773–6798		**+++**	**+**	**-**	**-**
33	4156	4156.0 (cecropin)^3^	**+**	**+**	**-**	**-**
34	4156		**-**	**+**	**-**	**-**

^1^ Based on the data of Table 4

^2^[36]

^3^[30]

### Transcriptomic analysis of *C*. *vicina* AMPs

In addition to the above mass spectrometric analysis of the maggots AMPs, their composition was further analyzed by transcriptome analysis as described in Materials and Methods. The transcriptome data are available at https://trace.ncbi.nlm.nih.gov/Traces/sra/sra.cgi?view=search_obj (submission number SRR5210297). The database of the mRNAs containing 1.1 million entries was screened using sequences of known *C*. *vicina* AMPs as a query. Table 6 summarizes major findings of the database analysis. The analysis revealed two sequences named here defensin 1 and defensin 2 identical to *C*. *vicina* [35] and *Lucilia sericata* [47] defensins. In addition, we found one sequence identical to cecropin and a wide variety of sequences with a high level of similarity with diptericins and proline-rich peptides previously isolated from the hemolymph of *C*. *vicina* [30, 35]. Poole of proline-rich peptides include 47 sequences with similarity level of 82–86% from the previously sequenced peptide (P<0.001). Diptericin-like peptides are present by 385 sequences with similarity level 70–82% to previously sequenced diptericin. Therefore, Table 6 contains only a small fraction of the likely diversity of diptericins and proline-rich peptides.

**Table 6:** Sequences of *C*. *vicina* AMPs determined by transcriptome analysis and peptide sequencing.

Peptide	UniProt ID	AA sequence	Information source	
			mRNA data base	Peptide sequencing
Defensin 1	C0HJX7	ATCDLLSGTGANHSACAAHCLLRGNRGGYCNGKAVCVCRN	**+**	**+**^1^
Defensin 2 (Lucifensin-II)	B3EWY5	ATCDLLSGTGIKHSACAAHCLLRGNRGGYCNGRAICVCRN	**+**	**-**
Cecropin	C0HJX8	GGWLKKIGKKIERVGQHTRDATIQGLAVAQQAANVAATAR	**+**	**+**^1^
Diptericin 1	C0HJX9	DSKPLNLVLPKEEPPNNPQTYGGGGGSRKDDFDVVLQGAQXEV…(N-terminal)	**-**	**+**^1^
Diptericin 2		QNKPFKLTLPKEEPKNLPQLYGGGGGSRKQGFDVSLGAQQKVWESQNKRHSVDNAGY	**+**	**-**
P-rich 1	C0HJY0	FVDRNRIPRSNNGPKIPIISNP…(N-terminal)	**-**	**+**^1^
P-rich 2		FVDRSRRPNSNNGPKIPIISNPPFNPMPDLPGRE	**+**	**-**
P-rich 3		FVDRSRRPNSNNGPKIPIISNPPFNPNARPAW	**+**	**-**
P-rich 4		IVDRSRRPNSNNGPKIPIISNPPLIQMPDLPG	**+**	**-**
Domesticin-like peptide		SRDARPVQPRFNPPPPKRERPIIYDAPIRRPGPKTMYA	**+**	**+**^2^

^**1**^ [30]

^**2**^ [36]

### FLIP7 cytotoxicity to human blood cells

Flow cytometry cytotoxicity assay based on the differential count of necrotic (propidium iodide positive, annexin V negative) and apoptotic (annexin V positive) cells incubated 12 hours in the cell culture medium supplemented with FLIP7 confirmed absence of the AMP complex direct toxicity to human peripheral blood lymphocytes and monocytes (Table 7). Number of necrotic lymphocytes and monocytes remained at the level of control variant. ANOVA test also revealed no significant effect on the amount of necrotic lymphocytes and monocytes in the whole range of concentrations according to P values represented in the table. The number of annexin V positive cells in the lymphocyte population did not reveal a significant FLIP7 impact as well. However, in the population of monocytes FLIP7 at concentration 10 mg/mL caused a significant increase of apoptotic cells number compared to control (P = 0.004). ANOVA test confirms a significant FLIP7 effect on the monocytes’ apoptosis in the studied concentration range.

**Table 7:** Numbers (mean ± standard error) of apoptotic (annexin V positive) and necrotic (propidium iodide positive, annexin V negative) human peripheral blood leukocytes per 100 gated cells after *in vitro* incubation with FLIP7.

FLIP7, mg/mL	Lymphocytes		Monocytes	
	Apoptotic	Necrotic	Apoptotic	Necrotic
0	2.5 ± 0.8	0.40 ± 0.136	26.8 ± 5.50	0.57 ± 0.180
0.5	2.2 ± 0.69	0.31 ± 0.139	27.5 ± 6.28	0.38 ± 0.103
5	2.2 ± 0.40	0.76 ± 0.263	40.4 ± 12.70	0.31 ± 0.077
10	3.5 ± 0.84	0.46 ± 0.183	71.7 ± 10.91	0.23 ± 0.049
P	0.817	0.384	0.009	0.207

## Discussion

Results of the study of *C*. *vicina* AMP complex presented in this article clearly demonstrate the complex activity against established antibiotic resistant biofilms formed by human pathogenic *E*. *coli*, *S*. *aureus* and *A*. *baumannii*. However, the biofilms’ resistance growth over planktonic cells’ level considerably depends on the bacterial species. The growth quantified by MBIC_90_/MIC ratio is negligible in *S*. *aureus* (from zero to twofold), moderate (three to tenfold) in *E*. *coli* and significant (18 to 51-fold) in *A*. *baumannii* strains. Nevertheless, increase of the biofilms resistance to the range of reference antibiotics in most cases was significantly higher (24 to 667-fold) than the complex values. FPL7 activity is mostly attractive in its MBEC/MIC ratio rather than on its absolute MIC value, in comparison to standard antibiotics. Another key advantage of the complex is its ability to block resistance development in Gram-negative bacteria [30]. Equally important is the fact that the complex is active against both planktonic and biofilm forms of bacteria. Since each bacterial infection is initiated by free-living cells and in most cases goes to form biofilms, it allows the complex to control different stages of disease development. The complex anti-biofilm activity involves two coordinated processes: cells killing and matrix degradation that occur at the same threshold concentrations of the complex and, therefore, may prevent the spread of surviving bacteria beyond the hotbed of infection after the destruction of the matrix.

To perform its anti-biofilm function, the complex operates with at least five fully sequenced peptides named in this paper as Calliphora defensin 1, cecropin, diptericin 1, P-rich 1 and domesticin-like peptides (Table 6). The same AMPs were identified in *C*. *vicina* using planktonic bacteria as a test system [30, 35,36]. Diptericin 1 had previously been only partially sequenced, now its complete sequence is available. Moreover, the data obtained demonstrated the presence of some additional anti-*E*. *coli* AMPs, which remain to be structurally characterized. Thus, the complex comprises at least 7 AMPs synthesized and accumulated in the maggots’ hemolymph in response to bacterial infection (Table 5). Sequenced peptides belong to four families of cationic AMPs: defensins, cecropins, diptericins and proline-rich peptides, respectively. Defensins, cecropins and proline-rich peptides are widely distributed and well-studied insect AMPs [17, 18]. Defensins are a group of AMPs containing α-helix/β-sheet elements coordinated by three disulfide bridges and known to be selectively active against Gram-positive bacteria. Cecropins are linear amphipathic α-helical AMPs selectively active against Gram-negative bacteria. Diptericins are members of glycine-rich AMP family selectively toxic to some Gram-negative Enterobacteria like *E*. *coli* by means of cell wall disruption [38]. *C*. *vicina* proline-rich peptides belong to the group of proline/arginine-rich AMPs, which are known to kill bacteria by damaging DNA and/or protein synthesis [39]. Thus, *C*. *vicina* AMP complex comprises three structurally distinct groups of cell wall disrupting AMPs targeted predominantly to the membranes of Gram-negative (cecropins, diptericins) or Gram-positive (defensins) bacteria and one group affecting intracellular targets (proline/arginine rich peptides).

Bioassays of the complex chromatographic fractions indicated that all found AMP families possess anti-biofilm activity of two types: cell killing (determined by TTC assay) and matrix destroying (determined by crystal violet assay). However, the proportions of these effects as well as activity against biofilms formed by different types of bacteria are characteristic for each family. Chromatographic fractions containing defensin 1 and P-rich 1 peptides provided the bulk of anti-staphylococcal activity and significant part of anti-*E*. *coli* activity. Cecropin, diptericin and domesticin-like peptide fractions as well as unidentified AMPs were selectively active against *E*. *coli* but not *S*. *aureus* biofilms. The diverse composition of the AMPs toxic to *E*. *coli* corresponds to the mass dissemination of Enterobacteria and related Gram-negative species in *C*. *vicina* natural habitats.

Analysis of the composition of *C*. *vicina* AMPs demonstrates sophisticated nature of this defense system. In reality it may be even more intricate. Transcriptome analysis revealed about 500 mRNA isoforms encoding sequences of all four AMP families. Of this amount, diptericins family contains not less than 385 mRNAs comprising motif of five glycine residues typical to insect diptericins and demonstrating 70–82% sequence similarity with previously sequenced *C*. *vicina* diptericin 1. Not all detected mRNAs lead to AMPs. For example, we have found in *C*. *vicina* mRNA encoding lucifensin-II, defensin from another blowfly species *Lucilia sericata* [47] but did not find the peptide itself. There is little doubt that many other AMP-encoding mRNAs also remain "silent" as it was demonstrated in some insects [29, 48]. Others may encode peptides having no antimicrobial activity. Nevertheless, we cannot exclude that the complex comprises more AMPs than found today.

AMPs are regarded as promising drug candidates for the treatment of biofilms [31–33]. According to data presented in this article, *C*. *vicina* defensins, diptericins, cecropins and proline-rich peptides can be added to this list. However, nature seems to be not in vain using multi-component complexes instead of single AMPs. In addition to the above-mentioned advantages, it allows the immune system to explore synergistic activity of various AMPs. The synergy is described repeatedly in the literature with regard to the combination of two [27–29] or three [49] AMPs. An analysis of more complex combinations remains a formidable task. No less important is the fact that the resulting synergistic combination must possess a number of other qualities to become a medicine. This makes the development of therapeutic AMP combinations extremely difficult. The study of the natural AMP complexes can be very helpful in solving this problem. Particularly promising from this point of view are AMP complexes of *C*. *vicina* and other blowflies living in environments with the highest concentration of human pathogenic microflora [25]. Besides the information about the structure and properties of the antimicrobial compositions withstood rigorous and impartial examination by natural selection, the larvae of *C*. *vicina* can supply the necessary components for their production. Methods of *in situ* and *in vitro* biosynthesis of *C*. *vicina* AMP complex make it technically and economically feasible [25, 36]. In future, this and similar natural AMP complexes can be applied in various fields of medicine, from routine bacterial infections to chronic inflammatory diseases and cancer. The most promising is their use for the treatment and prevention of diseases caused by antibiotic-resistant biofilms.
